# *Peperomia pellucida* leaf extract as immunostimulator in controlling motile aeromonad septicemia due to *Aeromonas hydrophila* in red hybrid tilapia, *Oreochromis* spp. farming

**DOI:** 10.14202/vetworld.2016.231-234

**Published:** 2016-03-05

**Authors:** S. W. Lee, K. Y. Sim, W. Wendy, A. K. Zulhisyam

**Affiliations:** 1 Faculty of Agro Based Industry, Universiti Malaysia Kelantan Jeli Campus, 17600, Jeli, Kelantan, Malaysia; 2 School of Fisheries and Aquaculture Sciences, Universiti Malaysia Terengganu, Kuala Terengganu, 21030, Terengganu, Malaysia

**Keywords:** *Aeromonas hydrophila*, immunostimulator, motile aeromonad septicemia, red hybrid tilapia

## Abstract

**Aim:**

This study was revealed the potential of *Peperomia pellucida* leaf extract as an immunostimulator agent in controlling motile aeromonad septicemia due to *Aeromonas hydrophila* in red hybrid tilapia, *Oreochromis* sp.

**Materials and Methods:**

In the present study, minimum inhibitory concentration (MIC) of *P. pellucida* leaf extract against *A. hydrophila* was determined through two-fold microbroth dilution method. The plant extract was screening for its active compound using a gas chromatograph mass spectrometer, and the effectiveness of *P. pellucida* leaf extract as an immunostimulator agent was evaluated. The experimental fish were fed with medicated feed at three different concentrations (25 mg/kg, PP-25; 50 mg/kg, PP-50; and 100 mg/kg, PP-100) of *P. pellucida* leaf extract for 1 week before they were intraperitoneally exposed to *A. hydrophila*. Enzyme-linked immunosorbent assay was carried out to determine the value of antibody response to *A. hydrophila* in fish from a group of fish that received medicated feed, and the percentage of total cumulative mortality of the experimental fish were observed at the end of the experiment.

**Results:**

The results showed that the major bioactive compound is phytol (40%), and the MIC value was 31.5 mg/L. The value of antibody response to *A. hydrophila* in fish from a group of fish which received medicated feed (PP-25, 0.128±0.014 optical density [OD]; PP-50, 0.132±0.003 OD; and PP-100, 0.171±0.02 OD) was found significantly higher (p<0.05) compared to fish did not receive medicated feed (0.00 OD). Whereas, percentage cumulative mortality of fish from all groups of fish received medicated feed (PP-25, 18.0±3.2%; PP-50, 18.2±2.8%; and PP-100, 17.7±1.8%) were found significantly lower (p<0.05) compared to a group of fish did not receive medicated feed (83.2±1.4%).

**Conclusion:**

The findings of the present study indicated the huge potential of *P. pellucida* leaf extract as natural immunostimulator agent for aquaculture uses.

## Introduction

Recently, aquaculture activities are expanding rapidly to generate more aquaculture production in fulfilling demand from the market. The expansion of aquaculture industry is due to the declining of fishery production and overexploitation of fishery product from the open sea. Subsequently, aquaculture is gearing toward intensification with the expectation to produce more fish. However, bacterial disease is recognized as a major constraint to the development of aquaculture industry. One of the bacterial diseases known as motile aeromonad septicemia (MAS) due to *Aeromonas hydrophila* was reported to cause devastation to tilapia fish farms.

*A. hydrophila* was reported found in many aquatic animals. For instance, Lee *et al*. [1] claimed that *A. hydrophila* was found in diseased Malaysian giant prawn, *Macrobrachium rosenbergii*. Other study was also reported that this bacterium was responsible to the mass mortality of ornamental fish in the aquarium shop. Based on the literature survey, *A. hydrophila* was found in Mantis Shrimp, *Squilla* sp., African catfish, *Clarias gariepinus*, American bullfrog, *Rana catesbeiana* [2], Asian seabass, *Lates calcarifer* [3], golden pompano, *Trachinotus blochii* [4], silver catfish, *Pangasius sutchi*, and red hybrid tilapia, *Oreochromis* sp. [5].

Traditionally, commercial antibiotic was used in fish health management. However, cases of antibiotic resistance have been alarming the industry due to the misuse and over use of the commercial antibiotics in aquaculture. Therefore, this study was performed to reveal the potential of *Peperomia pellucida* leaf extract as an alternative antimicrobial agent for aquaculture uses.

## Materials and Methods

### Ethical approval

The study was conducted following approved guidelines of the Institutional Animal Ethics Committee.

### Bacterial isolate

*A. hydrophila* isolated from diseased red hybrid tilapia, *Oreochromis* sp., at commercial farms in Kelantan, Malaysia was used in the experiment. The bacterial isolate was cultured using brain heart infusion broth (Oxoid, England) for 18 h at room temperature. The bacterial pellet was harvested by centrifugation at 13,500 rpm for 10 min. The harvested bacterial pellet was washed twice using physiological saline, and the concentration of the bacterial isolate was adjusted to 10^9^ colony forming unit/mL for challenged by intraperitoneal injection of 100 µl of each inoculum, at a dose causing 50% mortality (LD_50_).

### Plant extraction

*P. pellucida* leaf extract was prepared immediately after bought from the local market. The plants were clean using tap water and subjected to oven dried for 24 h at 40°C. *P. pellucida* leaf was extracted according to Lee *et al*. [6]. The extract was then stored at −20°C for further use.

### Minimum inhibitory concentration (MIC) values determination

MIC values were determined using two-fold micro broth dilution method in 96-wells microtiter plate format. The bacterial suspension was prepared as described above. Bacterial suspensions were inoculated into wells of a microtiter plate in the presence of the plant extract with concentration start from 0.15 to 31.5 mg/L and kanamycin as the positive control [7]. The growth of tested bacterial, *A. hydrophila*, was checked after 24 h (s) incubation via enzyme-linked immunosorbent assay (ELISA) reader (Bio-Rad, USA) at 540 nm. MIC value is determined as the lowest concentration of antimicrobial agent inhibits the visible growth of the inoculated bacteria [8].

### Determination of plant extracts chemical composition

The chemical composition of the plant extract was carried out as described by Lee and Wendy [9]. The chromatographic procedure was performed using a gas chromatograph mass spectrometer QP2010-GC-MS (Shimadzu, Japan) with autosampler. The sample was diluted 25 ti mes with acetone and 1 µl of sample was injected into a column. A fused silica capillary column HP5-MS (30 m × 0.32 mm, film thickness 0.25 µm) was used. Helium was the carrier gas, and a split ratio of 1/100 was used. The oven temperature used was maintained at 60°C for 8 min. The temperature was then gradually raised at a rate of 3°C per min to 180°C and maintained at 180°C for 5 min. The temperature at the injection port was 250°C. The components of the test solution were identified by comparing the spectra with those of known compounds stored in the internal library.

### Medicated feed

The fish pellets (Cargill, Malaysia) were purchased commercially before they were mixed with crude extract of *P. pellucida* leaf. Medicated feed was prepared in three different concentrations of *P. pellucida* leaf extract at 25 mg/kg, PP-25; 50 mg/kg, PP-50; and 100 mg/kg, PP-100. In the preliminary toxicity test, crude extract of *P. pellucida* showed adverse effect on the fish when given at concentrations of 150 mg/kg and above. The extract was coated onto fish pellet at a desired concentration and oven dried at 30°C for 24 h. The prepared fish pellet was then kept at −20°C for further use.

### Efficacy of medicated feed experiment

The antimicrobial agent efficacy test was carried out to determine the effectiveness of *P. pellucida* leaf extract in preventing and controlling MAS in red hybrid tilapia due to *A. hydrophila*. A total of 15 groups of fish, where each group contains 10-15 fish were maintained in 20 L aquaria. Six groups of fish were used as control, where each three groups served as negative and positive control, respectively. Nine groups of fish were used in the treatment using three different concentration of *P. pellucida* leaf extract (25 mg/kg, PP-25; 50 mg/kg, PP-50; and 100 mg/kg, PP-100), with triplicates for each treatment. The experimental fish were given medicated fish pellet at 2% body weight of fish a day for 1 week before the fish were exposed to *A. hydrophila* by intraperitoneal injection. The mortality of the infected fish was observed and recorded for 4 weeks. Simultaneously, the medicated and unmedicated fish pellet was continuously given to the fish for 4 weeks. Fish from each treatment was randomly sampled for weekly ELISA. The calculation of the fish mortality as shown below:


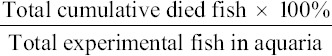


### Indirect ELISA

ELISA was carried out as described by Shelby *et al*. [10] with some modification. Briefly, fish were bled from the caudal vein and the blood was collected into a micro-centrifuge tube. The blood was then allowed to clot for 1 h at 25°C. The fish serum was harvested through centrifugation at 300 g and stored at −80°C for further use. The MAS antigen was prepared by a whole cell dilution of *A. hydrophila* with carbonate buffer to 500 µg/mL. A 100 µL of MAS antigen was added to each well of the microtiter plate for 1 h at 25°C. The wells were then blocked with 3% bovine serum albumin (Sigma, USA) for 1 h at 25°C. After the incubation period, the wells were washed 5 times with phosphate-buffered saline (PBS) plus teewn-20 (PBS-T). A volume of 100 µL of a serum sample (1 µL of serum diluted in 999 µL of PBS-T) were added to three replicate wells of the plate followed by 30 min incubation at 25°C. The wells were then washed 3 times with PBS-T. After washing, 100 µL of goat anti-tilapia immunoglobulin serum (diluted 1: 5000 in PBS-T) was added to the wells followed by 30 min incubation at 25°C. Again, after 3 times washing with PBS-T, 100 µL of rabbit anti-goat peroxidase conjugate (diluted 1: 5000 PBS-T) was added into the wells. Finally, the wells were washed again with PBS-T followed by the addition of 100 µL of *o*-phenylenediamine in urea-peroxide buffer into each well. The ELISA reaction was stopped at 15 min by adding 50 µl of 3 M H_2_SO_4_. The optical densities (OD) of the reactions were read with the microplate reader (Bio-Rad, USA) at 490 nm. Negative controls consisted of wells coated with antigen and no sample serum and wells with no antigen and a serum sample. The control reactions gave an optical density reading at 0.04 or less.

### Statistical analysis

Statistical differences between mortality and ELISA values were analyzed with one-way analysis of variance using Tukey *post-hoc* multiple comparison tests at 5% of a significant level.

## Results and Discussion

The results showed that the major bioactive compound is phytol (40%), and the MIC value was 31.5 mg/L. The value of antibody response to *A. hydrophila* in fish from a group of fish which received medicated feed (PP-25, 0.128±0.014 OD; PP-50, 0.132±0.003 OD; and PP-100, 0.171±0.02 OD) was found significantly higher (p<0.05) compared to fish did not receive medicated feed (0.00 OD). Whereas, percentage of cumulative mortality in fish from all groups of fish received medicated feed (PP-25, 18.0±3.2%; PP-50, 18.2±2.8%; and PP-100, 17.7±1.8%) was found significantly lower (p<0.05) compared to a group of fish did not receive medicated feed (83.2±1.4%) (Table-1).

**Table-1:** Cumulative mortality (%) of fish of the present study.

Treatment	Cumulative mortality (%)
Control	83.2±1.4
PP-25	18.0±3.2
PP-50	18.2±2.8
PP-100	17.7±1.8

In the present study, the major compound of *P. pellucida* leaf extract was phytol (39.12%) followed by 2-naphthalenol, decahydro- (27.10%), hexadecanoic acid, methyl ester (16.12%) and 9,12-octadecadienoic acid (Z,Z)-, methyl ester (17.66) (Table-2). However, none of the identified compounds in the present study were reported by Bayma *et al*. and Xu *et al*. [11,12], which may due to a different approach in characterizing chemical compounds in the plant extract. Previous studies have revealed the antimicrobial activity of *P. pellucida* leaf extract against various types of bacteria. For instance, Lee *et al*. [7] showed that the major compound of *P. pellucida* leaf extract is phytol which claimed to be responsible to the antimicrobial and antioxidant activity of the plant. This statement is supported by the study of Kumar *et al*. [13], where the study claimed that phytol is an important diterpene and possessed both antimicrobial and anticancer activities. In the literature, many studies were exploring the benefits of *P. pellucida* leaf extract. Nwokocha *et al*. [14] reported that *P. pellucida* was used as an anti-hypertensive remedy. However, none of the study revealed the potential of *P. pellucida* leaf extract as immunostimulator agent for aquaculture uses. Hence, this is the first report on the potential of *P. pellucida* leaf extract as an alternative of commercial antibiotic in fish health management.

**Table-2:** Compound composition of *P. pellucida* leaf extract.

Compound	Composition (%)
Phytol	39.12
2-Naphthalenol, decahydro-	27.10
Hexadecanoic acid, methyl ester	16.12
9,12-Octadecadienoic acid (Z, Z)-, methyl ester	17.66
Total	100

*P. pellucida=Peperomia pellucida*

Many studies have reported antimicrobial activity of plant against pathogenic bacteria from aquaculture sites. For example, *Allium sativum* extract [8], *Cymbopogon nardus* essential oil [9], *Andrographis paniculata* leaf extract [15], *Michelia champaca* seed and flower extracts [16], *Syzygium aromaticum* flower bud [17], *Citrus microcarpa* [18], *Murdannia bracteata* leaf extract [6], and many more. However, the mentioned studies were focus only on the *in vitro* antimicrobial activity of the plants. On the other hand, this study highlighted the mechanism and mode of action of the *P. pellucida* leaf extract in stimulating the immune system of the fish against infection of MAS due to *A. hydrophila*. In the near future, more immunological assays should be carried out to support the findings of the present study. For instance, immunological assays such as phagocytosis assay and chemiluminescence assay where these assays may give information on humoral and specific immune systems of fish and the response of the fish to pathogens [19].

## Conclusion

Based on the finding of the present study showed the huge potential of *P. pellucida* leaf extract as immunostimulator in controlling motile septicemia motile (MAS) due to *A. hydrophila* infected in red hybrid tilapia, *Oreochromis* sp. Hence, we proposed that this plant extract can be incorporated in the fish feed to manage fish health. However, further study should be carried out in the near future before we can come to a conclusion.

## Authors’ Contributions

SWL and KYS designed and supervised the study. SWL, WW, and AKZ conducted a study and analyzed the data. All authors contributed in the draft, revision, and approval of the final manuscript.
